# *In silico* evidence of signaling pathways of notch mediated networks in leukemia

**DOI:** 10.5936/csbj.201207005

**Published:** 2012-11-19

**Authors:** Kaiser Jamil, Archana Jayaraman, Raghunatha Rao, Suryanarayana Raju

**Affiliations:** a Centre for Biotechnology and Bioinformatics, School of Life sciences, Jawaharlal Nehru Institute of Advanced Studies (JNIAS), 6th Floor, Budha Bhawan, M.G. Road, Secunderabad 500003, Andhra Pradesh, India; b Oncology Department, Nizams Institute of Medical Sciences ( NIMS), Panjagutta, Hyderabad 500082, Andhra Pradesh, India

**Keywords:** Notch, protein interactors, STRING database, Leukemia, ALL, Oncomine

## Abstract

Notch signaling plays a critical role in cell fate determination and maintenance of progenitors in many developmental systems. Notch receptors have been shown to be expressed on hematopoietic progenitor cells as well as to various degrees in peripheral blood T and B lymphocytes, monocytes, and neutrophils. Our aim was to understand the protein interaction network, using Notch1 protein name as query in STRING database and we generated a model to assess the significance of Notch1 associated proteins in Acute Lymphoblastic Leukemia (ALL). We further analyzed the expression levels of the genes encoding hub proteins, using Oncomine database, to determine their significance in leukemogenesis. Of the forty two hub genes, we observed that sixteen genes were underexpressed and eleven genes were overexpressed in T-cell Acute Lymphoblastic samples in comparison to their expression levels in normal cells. Of these, we found three novel genes which have not been reported earlier- *KAT2B*, *PSEN1* (underexpressed) and *CDH2* (overexpressed).These three identified genes may provide new insights into the abnormal hematopoietic process observed in Leukemia as these genes are involved in Notch signaling and cell adhesion processes. It is evident that experimental validation of the protein interactors in leukemic cells could help in the identification of new diagnostic markers for leukemia.

## Introduction

Leukemia is an aggressive blood cancer, predominately diagnosed in children. It occurs when one lymphoblast, an immature white blood cell, turns malignant, multiplying uncontrollably and spreading rapidly throughout the body. If left untreated, the disease can be fatal in a few weeks. Unregulated proliferation of lymphoblasts is associated with deregulation of cell division and differentiation pathways. One of the key pathways involved in the regulation of cell differentiation and proliferation is the Notch signaling pathway and deregulation of the genes in this pathway may lead to uncontrolled proliferation observed in cancers such as Leukemias. Cancer related events are not just one gene signaling/defects, but multiple events which turn on the cells in synchrony to produce tumors, like clonal expansion. Therefore our intention was to understand the signaling network of Notch gene to understand how it promotes leukemogenesis which is not known until now.

The Notch signaling pathway, first described in *Drosophila* and *Caenorhabditis elegans*, is involved in the regulation of many developmental cell fate decisions. In Drosophilia, Notch interaction with its cell-bound ligands, Delta and Serrate, initiates an intercellular signaling pathway which plays a central role in development. In humans though homologues of the Notch-ligands have been identified, precise interactions and interplay between the ligands and the Notch homologues remain to be determined [2].

Mammalian Notch was first identified in 1991 in a translocation that brought the *Notch1* gene under the transcriptional control of the T-cell receptor β locus in a T-cell acute lymphoblastic leukemia [3]. The role of Notch in T-ALL was elucidated by Weng et al. [4], who demonstrated that over 60% of human T-ALL patients carried activating mutations in the *Notch1* gene. This observation led to further studies regarding the role of Notch in leukemia development. A report by Rosati et al. [5] implicated *Notch1* and *Notch2* signaling in B-cell chronic lymphocytic leukemia (B-CLL) and suggested a different mechanism for Notch activation than that observed by Weng et al. [4]. Yet, a clear understanding of the specific Notch receptors and ligands involved in this process, and the mechanisms of Notch action, are still lacking. Based on the various observations of Notch signaling pathway we decided to find out the players in notch signaling pathway. Hence the aim of our study was to construct a protein interaction network with Notch1protein as the query protein.

## Methods, Databases /Software

To decipher the associated protein interactors with Notch1 we designed a protocol, as described here, to record the observed interaction with other molecules which can help decipher which interactions could be associated with proliferative mechanisms. Further, we tried to assess the importance of the most highly connected proteins from this study by analyzing their clusters using bioinformatics database STRING [6] which was used to construct and retrieve protein interaction network. This database derives a high-throughput experimental data from a wide range of sources and analyses the co-expression of genes computationally and uses a scoring framework and outputs a single confidence score per prediction. This confidence score is a measure of reliability of the predicted interactions and a high score indicates that the predicted interactions are also replicated in KEGG database. Thus, we retrieved the Notch1 protein interaction network with a high confidence score from the STRING database and we analyzed the network to predict novel genes which may contribute to leukemogenesis.

### Construction of Protein-Protein Interaction (PPI) Network

To infer the interactions of NOTCH1 with other proteins, we used STRING database v9. STRING (Search Tool for the Retrieval of Interacting Genes, available at: http://string-db.org/ [6]) is a database of functional associations that have been pre-computed and derived from a wide range of sources such as high-throughput experimental data, literature and database mining, analyses of co-expressed genes and computational predictions. The database interactions are based on a scoring framework and the output interactions have a single confidence score per prediction. For our study, we queried the database using NOTCH1 as protein query name and selected the interactions available for *Homo sapiens*. We grew the interaction network to obtain 201 protein interactors. The database further allows the user to delineate interactions based on three levels of confidence scores- low (0.4 which represents 20%), medium (0.7 more than 50%) and high (0.9 greater than 75%). This score indicates the probability that a link predicted between two proteins is replicated in the KEGG database. To refine the quality of the NOTCH1 network, we selected only those interactions to be displayed that have a confidence score greater than 0.9 which indicates that the predicted network is highly reliable.

A better understanding of the network proteins and their interactions was obtained by grouping molecules which share some degree of similarity in terms of functional association. STRING database allows the user the choice of two clustering algorithms- MCL (Markov clustering) and k-Means Clustering. Further, the database allows the user to define the number of clusters. In the current study, we applied k-means clustering algorithm [7] as it is computationally fast, which is based on Adjacency matrix and delineates data into pre-defined number of clusters. We specified the number of clusters to be 10 based on the rule of thumb k= √(n/2), where n is number of nodes (protein interactors) in the cluster. The resulting clusters were separated manually for better visual representation and understanding.

### Gene Ontology (GO) Analysis

Enrichment analysis for the protein interactors in this network was performed using WebGestalt, WEB-based GEne SeT AnaLysis Toolkit (available at http://bioinfo.vanderbilt.edu/webgestalt/) [8], which is a data mining system that allows the user to explore and visualize biological information related to the data set. The protein interactors list was loaded onto the interface and analysis was performed with reference set selected as human genome. A Hypergeometric test was used to evaluate the statistical significance of the enrichment analysis. Benjamin and Hochberg method was used as the model for multiple test adjustment to check false discoveries. A *P*-value of 0.05 and a minimum of two genes per category were used as a cut-off to obtain significant enrichments.

### Analysis of the topology of Protein Protein Interaction (PPI) data, hub proteins, meta-analysis with expression data

The Notch1protein-protein interaction network was downloaded as an xml file from STRING database which contains data in Protein Standards Initiative (PSI) format. This file was imported into Cytoscape software [9] which is an open source Network visualization software with additional plugins for analysis of the network data. The Notch1 network was analyzed using Network Analyzer plugin [10] to obtain the topological properties of the interaction network. The topological properties include node degree distribution, average clustering coefficient, diameter, path length [11]. In a biological network, a node is any biological molecule and an edge indicates the interaction between two nodes.

Degree distribution: The degree of a node in a biological network is the number of interactions it forms. The degree distribution P(k) indicates the probability that a given node has exactly k interactions. For scale free networks the degree distribution follows power law i.e P(k)∼k^-γ^, wherein γ is the degree exponent [11].

Characteristic path length: The characteristic path length is the expected distance between two nodes [11].

Diameter: The diameter of the network is the maximum of the shortest distance between two nodes [11].

Clustering coefficient: Clustering coefficient indicates the propensity of nodes to cluster to form groups [11].

Further, to assess which genes in the network form hubs, cytoHubba plugin [12] was utilized with confidence value set to edge attribute and the ranking methods included degree and betweenness. Hubs are nodes with the highest number of interactions. The degree method was chosen as it indicates the importance of a particular node on the network. Betweenness centrality is a measure of influence of a particular node on the flow of information in the network [13, 14]. The cut off value for the top nodes was 50 proteins. The resulting list of 50 proteins from degree ranking method and 50 proteins from betweenness ranking method were compared and the proteins common to both lists (degree and betweenness) were utilized for further analysis. These common proteins were selected since they occur in both hub lists and hence may be more significant for analysis.

### Oncomine database for Microarray analysis

In order to assess the importance of these genes in Acute Lymphoblastic Leukemia, we analyzed the difference in the expression levels of the genes encoding these proteins in leukemic cells when compared to normal cells. Oncomine database is a comprehensive cancer microarray expression database wherein the expression data from the various studies are log-transformed and median-centered for each array [15]. The database allows the user to perform various analyses, in the form of filters, on the deposited expression data based on the user requirement. For the current study, the primary filters Cancer Type and Analysis Type were used to retrieve only those studies pertaining to T-cell Acute Lymphoblastic Leukemia wherein the database performed a differential analysis of the expression level of the genes in normal state vs. disease state. This showed two relevant datasets, Andersson et al. [16] and Haferlach et al. [17]. Each of the common hub genes was queried to assess their expression levels in these two studies. The database assigns a gene rank to each gene which pertains to the rank of that gene in a particular dataset based on its p-value. The p-value is calculated by the database based on Student's t-test statistic and indicates difference between the groups specified for differential analysis. The presence of the gene in both studies and the gene rank were used as parameters to assess the significance of the genes in T-cell Acute Lymphoblastic Leukemia.

## Results

### PPI Network and GO Analysis

**Figure 1:**
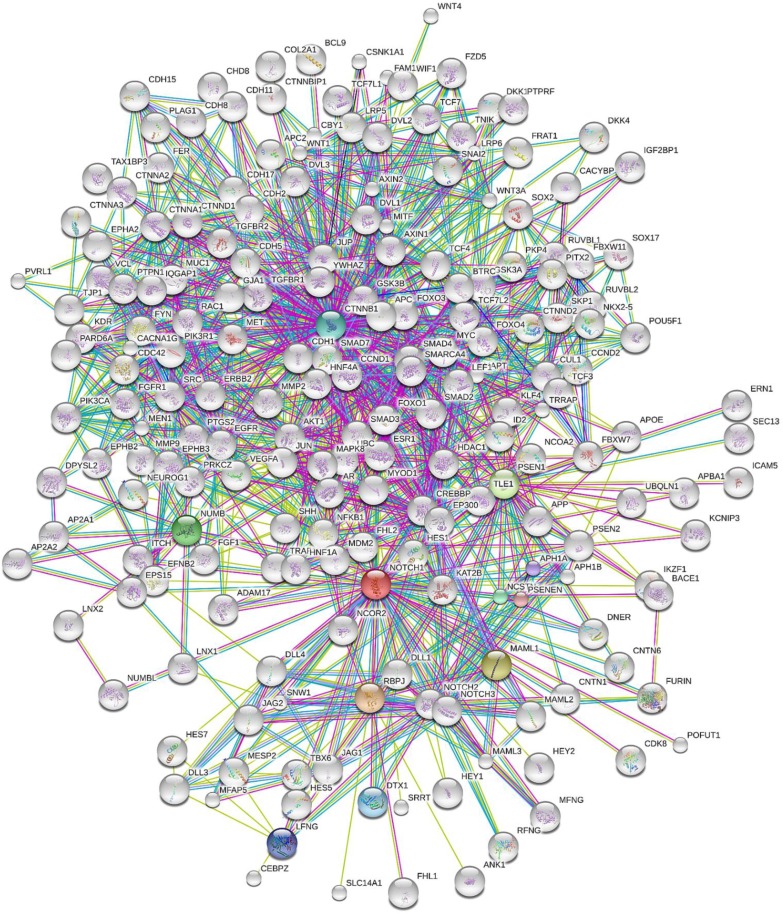
Notch1 Protein-Protein Interaction Network

The biological network derived from STRING database using Notch1 as query was observed to be a dense network comprising 201 interacting proteins. The most highly connected protein interactions seemed to be situated at the centre while Notch1 and its interacting proteins were observed to be located at the periphery. A few of the other protein interactors were also located at the periphery (Figure 1).

The gene ontology (GO) enrichment analysis results were represented in the form of a directed acyclic graph with the most significant terms highlighted in red. The biological process GO graph is shown in Supplementary Figure S1. Some of the important biological processes in which the genes encoding these proteins participate include signaling pathways such as Wnt and Notch, regulation of cell differentiation, cell fate commitment and regulation of metabolic processes. KEGG Pathway enrichment analysis revealed the proteins were involved in many pathways, important among them being Notch, TGF-beta, ErbB, MAPK, Jak- STAT, Wnt, T cell receptor and B cell receptor pathways. Some of the important pathways are shown in Table 1.

**Table 1:** Some important pathways identified through KEGG Pathway enrichment analysis

Pathway	No. of protein interactors
Notch signaling pathway	35
Wnt signaling pathway	45
TGF-beta signaling pathway	13
Cell cycle	14
MAPK signaling pathway	15
ErbB signaling pathway	10
Hedgehog signaling pathway	8
T cell receptor signaling pathway	8
B cell receptor signaling pathway	7
Jak-STAT signaling pathway	8

**Figure 2:**
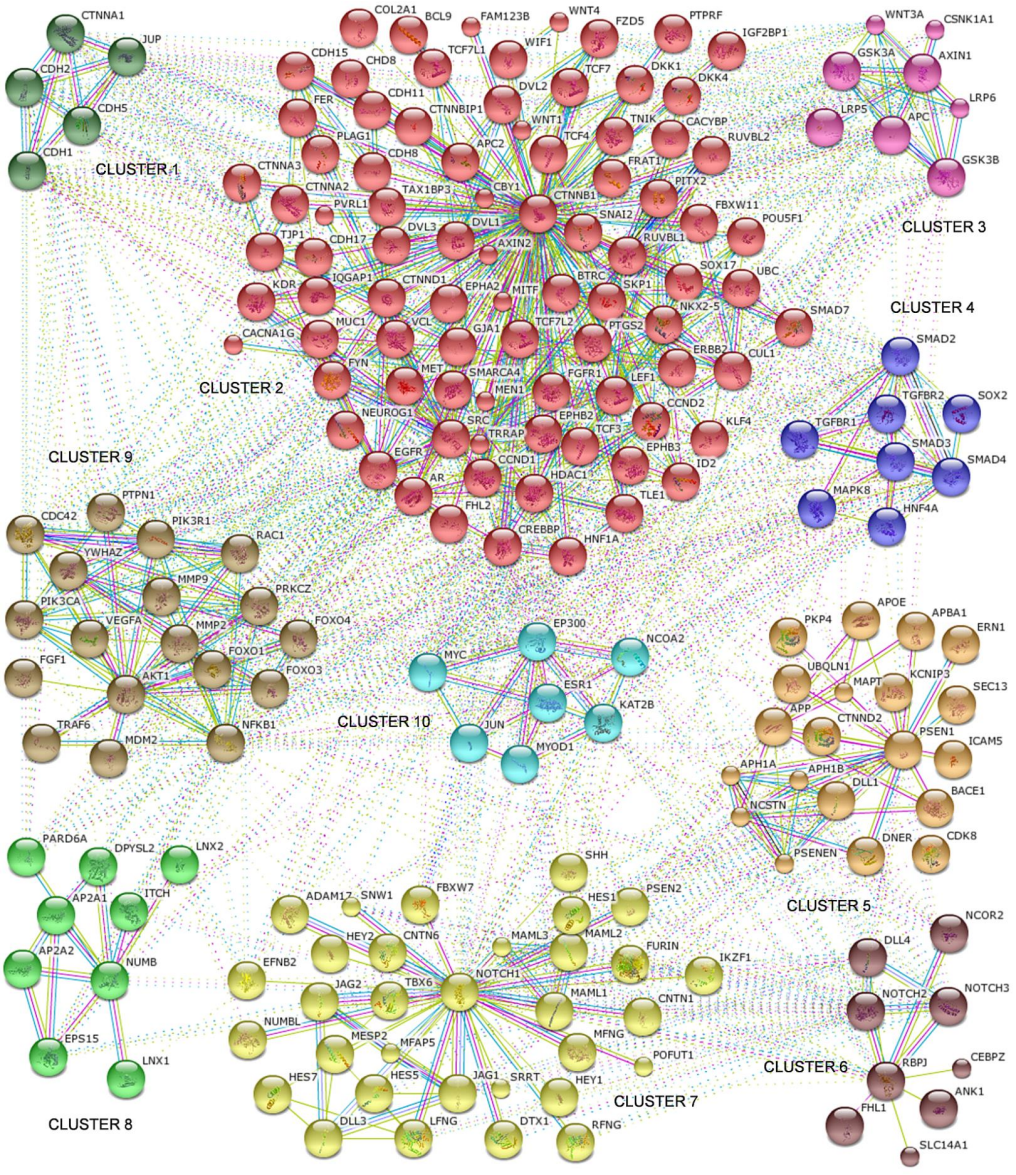
Notch 1 PPI network differentiated into 10 k-means clusters

To infer a better understanding of the interactions, the network was divided into ten clusters using k-means clustering option provided by the STRING database (Figure 2). The clusters were composed of densely connected protein interactors, mostly sharing either similarity in function or occurrence in the same pathway. Many of the interacting proteins were also connected with molecules in other clusters, probably indicating that they share functional associations with the other clusters, which might also represent that these molecules play key roles in several diverse pathways.

### Clusters obtained as shown in Figure 2

Cluster 1 contains proteins such as cadherins and cadherin associated proteins that are active in the process of cell adhesion. The biggest cluster, cluster 2, was centered around CTNNB1 molecule and contains proteins with diverse functions such as regulation of cell adhesion, Wnt signaling, cell proliferation, essential cell cycle proteins like CCND1,CCND2 and growth factors.

The protein interactors in cluster 3 are key participants of the Wnt signaling process. Wnt signaling has been reported to play an essential role in the hematopoietic development process. The fourth cluster is made of proteins that are important to TGF-beta mediated signaling process. This signaling is involved in processes such as proliferation, apoptosis, differentiation and migration, which are necessary for normal growth and development of an organism.

Some of the proteins in cluster 5 such as DLL1, DNER, and PSEN1 are important in Notch mediated signaling pathway. The other proteins involved in this process were adhesion proteins transport proteins and some others that were associated with gamma secretase complex.

The Notch1 cluster, which forms the seventh cluster, is a tightly connected cluster of 32 molecules. This cluster included molecules that were a common part of the Notch signaling network such as Notch binding ligands JAG1, JAG2, DLL3; activators of Notch pathway such as ADAM17; FBXW7 which are involved in the degradation of intracellular Notch; transcriptional coactivators of Notch such as MAML1-3; downstream effector of Notch signaling such as HEY1; targets such as HES1 and HES5 and transcriptional regulators such as IKZF1.

The molecules in the above cluster seem to be highly connected to cluster 6, which comprises proteins such as RBPJ (an important mediator of Notch signaling), Notch 2 and 3, DLL4 that are also integral parts of the Notch signaling pathway.

The protein interactors of cluster 8 are involved in functions such as cell migration, protein transport and cell growth and development.

Cluster 9 had fewer proteins of the PI3K- PI3K–PTEN signaling network pathway such as PIK3R1, AKT and the FOXO family of proteins. MDM2 and NFKB1 proteins are also part of this cluster. Most of the proteins in this cluster are involved in regulation of apoptotic mechanisms. MDM2 is an essential regulator of p53, and plays a vital role in p53 mediated apoptosis. NFKB1 plays an important role in various processes including cell differentiation, cell growth and apoptosis processes. Hence, the molecules in this cluster are crucial for the proper functioning of the cell cycle process and for differentiation.

Cluster 10 is composed of transcriptional regulators such as MYC, EP300, JUN, KAT2B that act as activators and transcription factors and are essential for the proper expression of several genes.

### Analysis of the topology of PPI data, hub proteins, meta-analysis with expression data

Analysis of the Network revealed that the Notch1 protein interaction network is a small world scale free network which follows power law (P(k)∼ k^-γ^) of node degree distribution with a degree exponent of 0.856 and R^2^ of 0.737, where R^2^ indicates the fitness of data points to the curve. The characteristic path length was calculated by the software to be 2.342 and the network diameter was found by the software to be 4.The clustering coefficient, which is a measure of cluster forming ability of a particular node, was found to be 0.520 [11].

The hub genes obtained from degree ranking method and betweenness method were compared to derive genes which are common to both and hence may play a role in the disease process. Forty two genes (Table 2) were observed to be common to both the degree and betweenness methods and this included the query molecule, Notch1. The expression level of each of these genes when queried in Oncomine database revealed that some of these genes were significantly overexpressed in the T-ALL samples while some were underexpressed. Only those genes whose expression has been reported in both of the studies used for the analysis were considered significant. Oncomine calculates the gene rank of each gene based on its p-value for each study and this gene rank is an indicator of the significance of the gene in a particular study. We considered only those genes whose cumulative gene rank from both the analysis was in the top 36% of all the over- or under-expressed genes in the studies. This step was incorporated to ensure that the change in expression of the genes was significantly different in the leukemic cells when compared to their expression in normal cells. The resultant gene list consisted of sixteen genes which were significantly underexpressed and eleven genes which were significantly overexpressed in relation to other genes in T-cell Acute Lymphoblastic cells in comparison to normal cells (Table 3). Most of the underexpressed genes are important tumor suppressors and/or play an essential role in the apoptotic process. The genes which are significantly overexpressed function in cell cycle progression, proliferation, cell survival, transcriptional regulation and development. These genes were queried in earlier studies on ALL to determine if their role in leukemogenesis has been established. While many of the genes in this list have been reported earlier in association with ALL [4, 18–28], some have not yet been studied in relation to the disease. These include the genes *KAT2B*, *CDH2* and *PSEN1* which have important biological significance in the hematopoiesis process due to their role in Notch signaling (*KAT2B* and *PSEN1*) and cell adhesion (*CDH2*) and their role in leukemogenesis has yet to be investigated. Three other genes from our study - *SMAD4*, *CTNNA1*, *RAC1*-have been reported in earlier studies but their role in T-cell leukemogenesis has not yet been elucidated.

**Table 2:** List of Common Hub proteins using degree and betweenness centrality ranking methods

S.No.	Protein	S.No.	Protein
1.	AKT1	22.	HDAC1
2.	APC	23.	HNF4A
3.	APP	24.	JUN
4.	AR	25.	JUP
5.	AXIN1	26.	KAT2B
6.	CCND1	27.	LEF1
7.	CDC42	28.	MYC
8.	CDH1	29.	MYOD1
9.	CDH2	30.	NFKB1
10.	CDH5	31.	NOTCH1
11.	CREBBP	32.	NOTCH2
12.	CTNNA1	33.	NOTCH3
13.	CTNNB1	34.	PSEN1
14.	CTNND1	35.	RAC1
15.	DLL1	36.	RBPJ
16.	DVL1	37.	SMAD2
17.	EGFR	38.	SMAD3
18.	EP300	39.	SMAD4
19.	ESR1	40.	SRC
20.	GSK3A	41.	UBC
21.	GSK3B	42.	VEGFA

**Table 3:** Hub protein encoding genes that have significant expression levels, observed in T-cell Acute Lymphoblastic Leukemia datasets of Andersson et al. (16) and Haferlach et al. (17) in Oncomine database

S.No.	Gene Symbol	Andersson et al. (16)			Haferlach et al. (17)		

		Gene Rank	p-value	Fold Change	Gene Rank	p-value	Fold Change

Underexpressed							
1.	APC	top 7%	1.99E-06	-4.782	top 21%	3.15E-08	-1.272
2.	APP	top 2%	4.68E-09	-19.629	top 1%	9.83E-64	-3.799
3.	AXIN1	top 27%	5.00E-03	-1.766	top 41%	3.70E-02	-1.607
4.	CCND1	top 27%	6.00E-03	-2.555	top 39%	1.90E-02	-1.08
5.	CDC42	top 3%	8.32E-08	-2.631	top 15%	2.44E-12	-1.249
6.	CDH1	top 18%	3.16E-04	-2.573	top6%	1.31E-28	-2.816
7.	CREBBP	top 1%	1.95E-09	-3.821	top 12%	1.66E-16	-1.408
8.	CTNNA1	top 22%	1.00E-03	-3.401	top 1%	4.41E-60	-3.627
9.	EP300	top 14%	5.14E-05	-1.732	top 34%	2.00E-03	-1.147
10.	KAT2B	top 2%	2.52E-08	-3.205	top 7%	4.59E-25	-2.279
11.	NFKB1	top 10%	3.40E-07	-29.14	top 5%	1.09E-19	-1.8
12.	NOTCH2	top 11%	1.93E-05	-5.629	top 18%	4.85E-10	-1.189
13.	PSEN1	top 2%	8.94E-09	-2.626	top 14%	5.38E-14	-1.338
14.	RAC1	top 42%	1.28E-01	-1.223	top 10%	3.25E-18	-1.438
15.	SMAD3	top 2%	6.69E-09	-10.45	top 27%	3.03E-05	-1.191
16.	VEGFA	top 33%	2.60E-02	-6.262	top 1%	1.25E-52	-3.364

**Overexpressed**							

1.	AKT1	top 16%	9.24E-05	2.159	top 26%	3.30E-06	1.224
2.	CDH2	top 30%	6.00E-03	3.89	top 4%	5.73E-32	2.648
3.	EGFR	top34%	1.40E-02	3.384	top 39%	5.00E-03	1.083
4.	GSK3B	top 31%	7.00E-03	1.398	top 13%	1.63E-14	1.364
5.	HDAC1	top 5%	2.38E-07	2.406	top6%	1.26E-24	1.542
6.	JUN	top 26%	2.00E-03	3.799	top 20%	1.12E-09	1.957
7.	LEF1	top 5%	3.97E-07	13.361	top 1%	8.79E-50	3.083
8.	NOTCH1	top 15%	6.77E-05	2.394	top 1%	5.05E-45	2.461
9.	NOTCH3	top20%	4.16E-04	11.153	top 2%	2.37E-38	3.021
10.	SMAD2	top 8%	2.25E-06	1.791	top 9%	1.41E-20	1.358
11.	SMAD4	top 2%	2.50E-02	1.276	top 37%	4.57E-42	2.024

## Discussion

**Figure 3:**
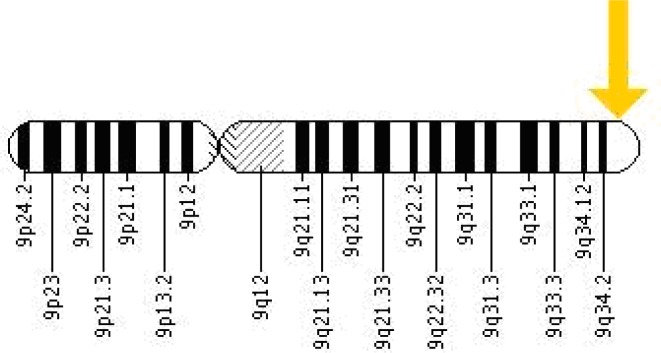
Chromosomal location of *Notch1* gene

The Notch family of genes, which in Mammals include the four transmembrane Notch receptor homologs (*Notch1,Notch2, Notch3, Notch4*), plays a key role in regulating many developmental differentiation processes including hematopoiesis during embryogenesis, T cell lineage commitment and maturation in the thymus and Marginal zone B cell development in the spleen [29]. The Notch signaling pathway involves many transcriptional regulatory processes [30]. These glycoprotein receptors consist of iterated structural motifs and an intracellular ankyrin-like repeat region that plays an important role in downstream signaling events. The Notch receptors bind to any of the five ligands, Delta-like 1, 3 and 4 and Jagged 1 and 2, which initiates the signaling pathway [31, 32]. Ligand binding to Notch protein leads to proteolytic cleavage and release of Notch intracellular domain (NICD) fragment which is the key signaling component [33]. Notch proteins are 300kDa ligand-activated heterodimeric receptors [1]. The *Notch 1* gene is located on the long (q) arm of chromosome 9 at position 34.3 (Figure 3).

### Disruptions in signaling due to mutations in Notch1

The Notch homologs and their ligands have been reported to be expressed in bone marrow, fetal liver, thymus, stromal cells indicating that they play an important role in the hematopoietic process [34]. *Notch1* consists of two key domains- PEST and the HD domains. Mutations in the PEST domain lead to deletion of this region, which normally regulates NICD degradation and hence leads to increased NOTCH1 activity. Mutations in the N- and C-terminal of the HD domain lead to ligand-independent Notch signaling. Thus alterations in this gene and its protein product lead to severe implications in the biological processes in which it functions [35]. Mutation in *Notch1* has been implicated in the pathogenesis of Leukemias. These alterations also affect the Notch signaling network leading to uncontrolled proliferations and disruptive differentiation processes which are characteristic of leukemias. Also, the genes/proteins involved in Notch signaling and other interactors of Notch may contribute to increased severity of disease process through deregulation of other pathways in which they participate. Among the Leukemias, Notch1 deregulation is more commonly observed in T-cell Acute Lymphoblastic Leukemia which may be due to the importance of Notch signaling pathway in T cell development and proliferation of committed T-lineage progenitor cells [36].

Studies have reported that mutations in *Notch1* prevent functioning of domains that are essential to prevent spontaneous activation of Notch. These mutations lead to increase in Notch signaling which in turn leads to deregulation of the signaling pathway. Mutations in *FBXW7* also promote increase in Notch signaling by supporting the stability of activated Notch1 [37]. Palomero et al. [38] have reported that Notch and MYC regulate interconnected transcriptional pathways which involve many common genes and which help in regulation of cell growth in T-ALL leukemic cells.

### Mechanism of Notch1 signaling

**Figure 4:**
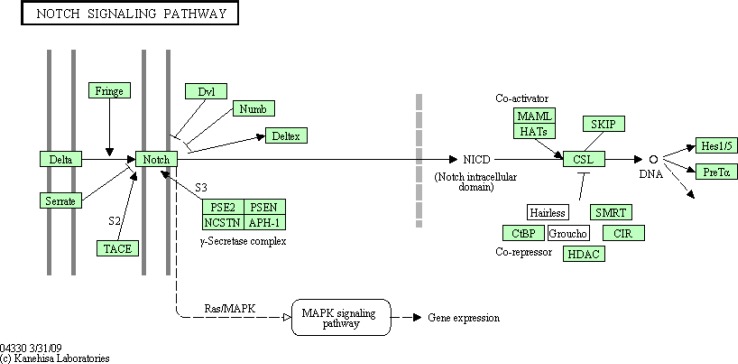
KEGG Pathway database map showing Notch signaling including proteolytic cleavage and translocation of Notch followed by transcriptional activation of Notch target genes

The Notch protein sits like a trigger spanning the cell membrane, with part of it inside and part outside. Ligand binding to the extracellular domain induces proteolytic cleavage with the release of the intracellular domain which enters the nucleus to alter gene expression [39] (Figure 4).

The Notch cascade involves the transmission of Notch signal to the cell nucleus with the help of the various Notch proteins, their ligands and intracellular proteins. The Notch/Lin-12/Glp-1 receptor family has also been reported to be implicated in cell fate specification during development in *Drosophila* and *C*. *elegans* [1].

Palomero et al. [40] showed that, in rare cases of Acute Myeloid Leukemia (AML), activated mutation in Notch1 contributed to hindrance in granulocytic differentiation process and led to maintenance of myeloid cells in immature phenotypic state. Other studies have implicated that alterations in the Notch ligand, JAGGED1, might contribute to the leukemogenesis process in AML [41]. A study by Chen et al. [42], in AML cells, reported that downregulation of Notch1 leads to a decrease in expression of PU.1/MCSFR, which leads to dysregulation in the myeloid cells. Notch signaling may also be involved in Chronic Myeloid Leukemia leukemogenesis through HES1, though this has not been experimentally validated [43]. Rosati et al. [5] suggest that Notch signaling plays a crucial role in survival and apoptosis resistance in B-Chronic Lymphocytic Leukemia cells with the cells expressing Notch1 and 2 and Jagged 1 and 2 which are not usually found in normal B cells. These studies highlight the importance of Notch signaling in the leukemogenesis process. Notch signaling is involved in the regulation of stem cell self-renewal in several systems. Meta analysis of genes and proteins helps provide insight into key processes regulating neoplastic and other dysregulated processes and also help in determination of therapeutic targets and development of therapeutic moieties as demonstrated through our previous studies [44–54].

In our study we present a different picture of Notch interacting players. We have observed that, of the clusters obtained, three clusters seem to be involved in mechanisms leading to neoplastic transformation. Two of the clusters are mainly involved in the Notch signaling network and hence alterations in the proteins in the cluster would affect differentiation and proliferation processes. The Notch 1 protein is also associated with molecules in the ninth cluster, many of whose proteins are involved in the PI3K-PTENpathway. This cluster consists of proteins that play a crucial role in control of cellular proliferation. Alterations in the proteins of this cluster and also changes brought about in Notch1 or its signaling network could cause deregulation of the proliferation processes leading to increased and uncontrolled proliferative signals, defects in differentiation process and to a block in the apoptotic process. Notch signaling has also been reported to participate in antiapoptotic mechanisms through p53 and NFκB pathway [55–58]. Deregulation of these mechanisms leads to increased deterrence of apoptosis which in turn prevents elimination of leukemic/ neoplastic cells.

The Notch1 cluster is also connected to cluster 4 through SMAD3, which along with the other proteins in this cluster, plays an important role in TGF-beta mediated signaling which helps in the regulation of cell cycle and apoptosis processes. Alterations in Notch1 might affect expression of SMAD3, hindering the cell cycle process and resulting in uncontrolled proliferation.

Many proteins in this network also participate in Wnt mediated regulation of hematopoietic development. Recent studies have reported that upregulation of this signaling pathway was observed in most of the Leukemias [59], highlighting the need to direct therapeutic approaches targeting this signaling pathway.

In this study, we observed that the protein interactors associated with disease seem to cluster together and to interact closely with other clusters that might also be implicated in disease pathogenesis. This observation is similar to reports from other studies wherein proteins involved in a particular disease tend to form a closely associated network [60]. We observed that proteins that play key roles in control of proliferation and differentiation are associated directly or indirectly with Notch1 or other molecules of the Notch signaling network. These associations might be indicative of interconnected pathways/ mechanisms whose deregulation and alterations might provide oncogenic signals. Understanding the interactions between proteins and/or genes is crucial as studies on cancer metasignature genes have implied that proliferation of cancer may involve cancer related genes from numerous pathways [61]. Though the external triggers for the leukemogenic signals are not yet clearly understood, analysis of the molecular pathways through which these signals proceed might help in developing more effective therapeutic strategies and in identification of better prognostic markers. Notch proteins regulate a broad spectrum of cell fate decisions and differentiation processes during fetal and postnatal development.

### Hub genes

Our study analyzed the protein interaction network data to identify hub proteins. We identified 50 hub proteins, through degree and betweenness ranking methods, each of which had numerous interactions within the network. On analysis of the expression levels of the genes encoding the common hub proteins, we observed that the genes that were associated with regulation of apoptosis and that have crucial tumor suppressor functions were underexpressed, which is characteristic of Leukemia and other cancers. We also found that the genes involved in various biological processes such as cell cycle, proliferation and gene expression were overexpressed. Through this analysis, we have identified three proteins- KAT2B, PSEN1 and CDH2- which are important hub proteins and whose expression levels were deregulated in T-cell Leukemic samples in the studies by Andersson et al. [16] and Haferlach et al. [17].

KAT2B is a transcriptional coactivator that plays a role in transcriptional regulation and inhibition of cell cycle progression. PSEN1 is involved in the proteolytic processing of membrane proteins such as Notch, TrkB, and APLP2 and whose expression has been reported in lymphocytes [62]. KAT2B and PSEN1, which were observed to be underexpressed, are important components of the Notch signaling network. Notch intracellular domain fragment (NICD) translocates to the nucleus and forms a complex with DNA binding protein CSL. This complex along with the help of co-activators MAML and KAT2B leads to the transcriptional activation of Notch target genes [63]. Gamma secretase complex which comprises PSEN1 and 2, NCSTN, APH-1 and PEN-2 cleaves Notch molecule at its transmembrane domain to give rise to Notch intracellular Domain fragment (NICD) [64] which translocates to the nucleus and leads to activation and propagation of Notch signaling. CDH2 is a calcium dependent cell adhesion protein. Studies by Wilson et al. [65] have shown that maintenance of balance between self-renewal and differentiation of hematopoietic stem cells by c-Myc occurs in N-cadherin dependent manner, specifically c-Myc associated differentiation of Hematopoietic stem cells and their subsequent movement is associated with CDH2 downregulation. The overexpression of CDH2 observed in T-cell Leukemia samples analyzed in our study and its role as a hub protein indicates that this gene needs to be investigated further using experimental procedures to assess its significance in Leukemogenesis.

In interaction networks, hubs are usually molecules that have the highest number of interactions in the network. Hub genes are thought to play crucial roles in cell processes and hence possess biological significance [66]. Several studies have been carried out to determine the importance of hub molecules in cellular processes and diseases. Batada et al. [67] have suggested that hub proteins play an essential role in rate of cell growth and are carefully regulated. Hub proteins have also been associated with high lethality in a study on yeast proteins [66]. Zotenko et al. [68] have proposed that hub proteins are essential as they are usually a part of a dense network of essential proteins which are needed to perform specific biological functions.

Jonson and Bates [60] have reported that generally cancer-related proteins have more interactions than non-cancer proteins. Goh et al. [69] report that though essential genes encode hub proteins, most of the disease genes are non-essential and are do not encode hub proteins. Though there are varied views regarding the significance of hub molecules in disease almost all the studies agree that genes that encode hub proteins have biological significance.

Expression of some of the other genes such as *SMAD4* [18], *CTNNA1* [19], *RAC1* [20] has been reported but their contribution to the leukemogenesis process has not been clearly understood.

In the expression datasets analyzed, *NFKB1* was observed to be underexpressed. This was also reported by Chang et al. [70] and they associate this alteration in expression to targeting of *NFKB1* promoter by the TAL1 oncoprotein. The underexpression of *CCND1* and *RAC1*, which are generally overexpressed in neoplasms, in the datasets under study might indicate that these genes were not associated with leukemogenesis in the patient samples used for the expression study. Although most cancers show high expression levels of *VEGF*, in some cancers such as benign forms of melanoma [71] and in cancers with high microsatellite instability [72] lower expression of *VEGFA* are associated with better prognosis. The underexpression of *VEGFA* in the T-ALL datasets examined may also be indicative of favorable prognosis in the T-ALL patients. Gottardo et al. [73] also reported downregulation of *Notch2* in their analysis of T-ALL and they associate this lowered expression to adverse outcome in the patients.

Though in majority of the cancers underexpression of tumor suppressor *SMAD2* has been reported, in a few cancers such as Pancreatic cancer and Gastric cancer, an overexpression of this protein has been observed [74, 75]. Researchers have attributed this increase to the shift in TGFβ from tumor suppressor to oncogene in advanced metastasis [75]. Shinto et al. [75] have proposed that since this protein is involved in activation of *SMAD2*, an increase in TGFβ may lead to a concomitant increase in *SMAD2* expression and thus contributes to increased cell proliferation through TGFβ receptor signaling pathway [75]. This mechanism may also be prevalent in T-ALL.

*SMAD4* is also a major tumor suppressor which is generally downregulated in cancers. Studies have reported *SMAD4* overexpression in Rhabdomyosarcoma cells and suggest that this upregulation is through *TGFβ1* [76]. A similar mechanism may also take place in T-ALL and contribute to TGFβ-mediated aberrant proliferation in these cells.

Thus, this study identified three unreported genes in leukemogenesis.

## Conclusion

The Notch intercellular signaling pathway is conserved evolutionarily and regulates interactions between adjacent cells. Protein interaction studies through network biology help provide an insight into possible disease pathways. The interactors associated with disease proteins might play key roles in the mechanism of disease and hence a study of protein interactions is essential towards understanding disease etiology. In our study, we observed that the Notch signaling proteins are closely associated functionally with proteins that are crucial in cell cycle, cell differentiation and apoptosis processes. Our study of the network of Notch1 protein interactors has highlighted the possible interactions through which Notch signaling might lead to uncontrolled proliferation of cells leading to neoplastic transformation resulting in Leukemia. The Notch1 interaction network shows that the protein interactors of Notch1 play an equally crucial and specific role in Notch mediated signaling. These interactions could signify that therapeutic approaches concentrating on specific Notch mediated targets and proteins that associate with Notch and help in its control of cell proliferation, might prove as effective targets towards preventing proliferation of leukemic cells. We have identified three genes-*KAT2B*, *PSEN1* and *CDH2*- which encode hub proteins and investigating their role in leukemogenesis might help in development of new therapeutic measures. It is predicted that this study could serve as a repository for selection of prognostic and therapeutic moieties involved in Notch mediated leukemia.

## Supplementary Material

Gene Ontology for Notch PPI network
