# Supplementary figures and images for: In silico evidence of signaling pathways of notch mediated networks in leukemia
Source: Comput Struct Biotechnol J. 2012 Nov 19;1:e201207005. doi: 10.5936/csbj.201207005 (PMC3962152; doi:10.5936/csbj.201207005)

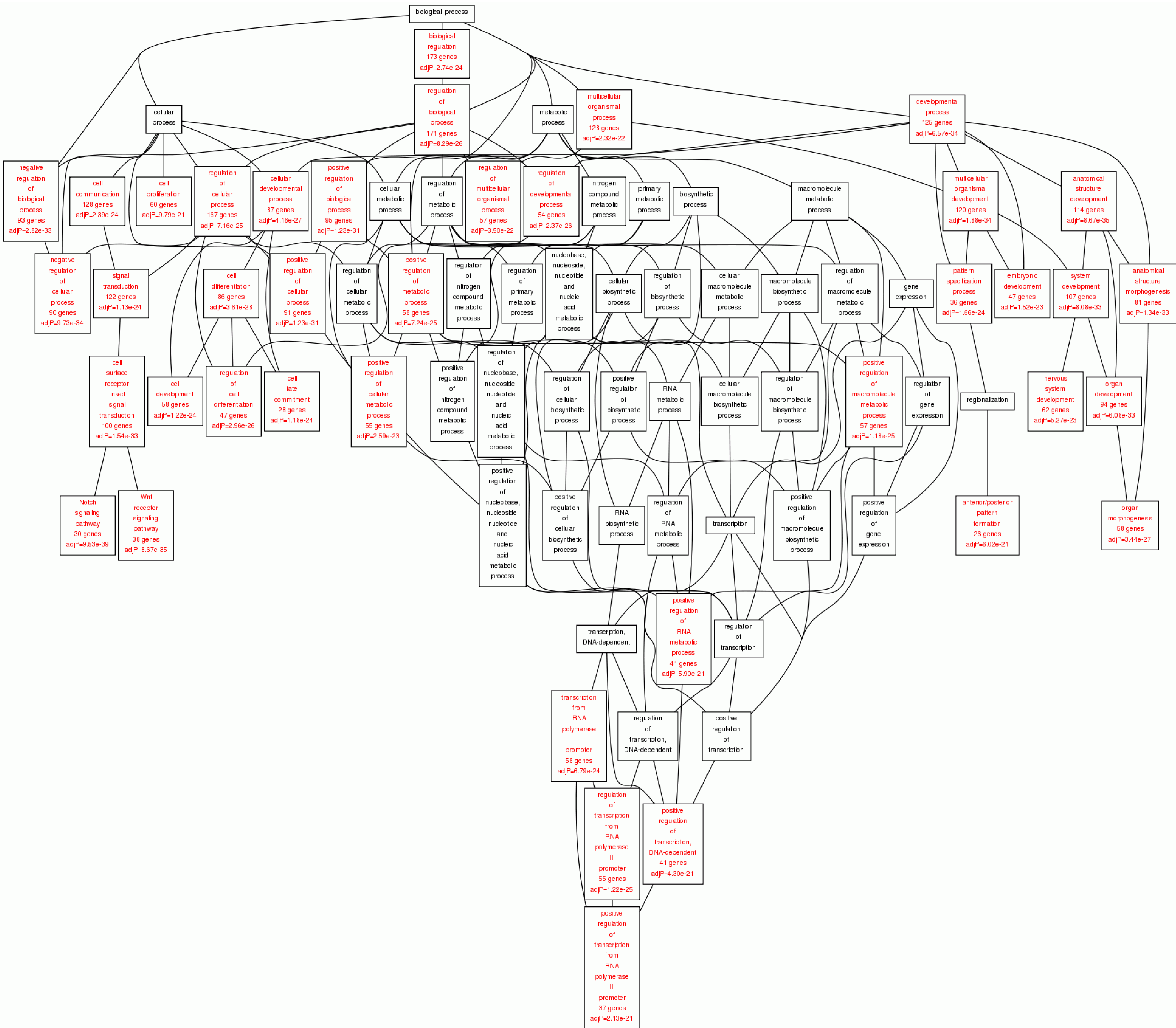

**Supplementary Figure S1.**  
Gene Ontology for Notch PPI network

Supplement: Gene Ontology for Notch PPI network [file CSBJ-1-e201207005_SM0001.pdf]
